# A new regulatory mechanism for bacterial lipoic acid synthesis

**DOI:** 10.1002/mbo3.237

**Published:** 2015-01-22

**Authors:** Huimin Zhang, Qixia Luo, Haichun Gao, Youjun Feng

**Affiliations:** 1 Center for Infection and Immunity, Department of Medical Microbiology and Parasitology, School of Basic Medical Sciences, Zhejiang UniversityHangzhou, Zhejiang, China; 2 Institute of Microbiology, College of Life Sciences, Zhejiang UniversityHangzhou, Zhejiang, China

**Keywords:** cAMP-receptor protein (CRP), LipA, LipB, Lipoic acid, lipoic acid synthesis, *Shewanella*

## Abstract

Lipoic acid, an essential enzyme cofactor, is required in three domains of life. In the past 60 years since its discovery, most of the pathway for lipoic acid synthesis and metabolism has been elucidated. However, genetic control of lipoic acid synthesis remains unclear. Here, we report integrative evidence that bacterial cAMP-dependent signaling is linked to lipoic acid synthesis in *Shewanella* species, the certain of unique marine-borne bacteria with special ability of metal reduction. Physiological requirement of protein lipoylation in *γ*-proteobacteria including *Shewanella oneidensis* was detected using Western blotting with rabbit anti-lipoyl protein primary antibody. The two genes (*lipB* and *lipA*) encoding lipoic acid synthesis pathway were proved to be organized into an operon *lipBA* in *Shewanella*, and the promoter was mapped. Electrophoretic mobility shift assays confirmed that the putative CRP-recognizable site (AAGTGTGATCTATCTTACATTT) binds to cAMP-CRP protein with origins of both *Escherichia coli* and *Shewanella*. The native *lipBA* promoter of *Shewanella* was fused to a LacZ reporter gene to create a chromosome *lipBA-lacZ* transcriptional fusion in *E. coli* and *S. oneidensis*, allowing us to directly assay its expression level by *β*-galactosidase activity. As anticipated, the removal of *E. coli crp* gene gave above fourfold increment of *lipBA* promoter-driven *β*-gal expression. The similar scenario was confirmed by both the real-time quantitative PCR and the LacZ transcriptional fusion in the *crp* mutant of *Shewanella*. Furthermore, the glucose effect on the *lipBA* expression of *Shewanella* was evaluated in the alternative microorganism *E. coli*. As anticipated, an addition of glucose into media effectively induces the transcriptional level of *Shewanella lipBA* in that the lowered cAMP level relieves the repression of *lipBA* by cAMP-CRP complex. Therefore, our finding might represent a first paradigm mechanism for genetic control of bacterial lipoic acid synthesis.

## Introduction

Lipoic acid (6,8-dithiooctanoic acid, thioctic acid, or R-5-(1,2-dithiolan-3-yl) pentanoic acid), is a type of two-sulfur inserted eight-carbon fatty acid derivative and acts as a coenzyme widespread in three domains of life (Perham 2000; Cronan et al. 2005). This covalently bound cofactor is required for aerobic metabolism of 2-oxoacids in *Escherichia coli* and C1 metabolism in plants like *Arabidopsis* (Perham 2000; Cronan et al. 2005; Engel et al. 2007). In *E. coli*, the three well-known enzymes whose activities require lipoylation, the post-translational modification, include PDH (pyruvate dehydrogenase), OGDH (2-oxoglutarate dehydrogenase), and GCV (glycine cleavage system) system (Cronan et al. 2005; Hermes and Cronan 2009). All the three enzyme systems possess such subunits (the E2 subunits of both PDH and OGDH, and the H protein of GCV system) that contain no less one lipoyl domains (LD) featuring with a conserved structure of around 80 residues long (Reche 2000; Cronan et al. 2005). Generally, a specific/conserved lysine residue on these LDs is attached by lipoic acid via an amide bond (Perham 2000). Therefore, it seems likely that lipoic acid facilitates shuttle of the activated reaction intermediates amongst the active sites of the lipoate-dependent multi-enzyme systems (Perham 2000; Cronan et al. 2005).

**Figure 1:**
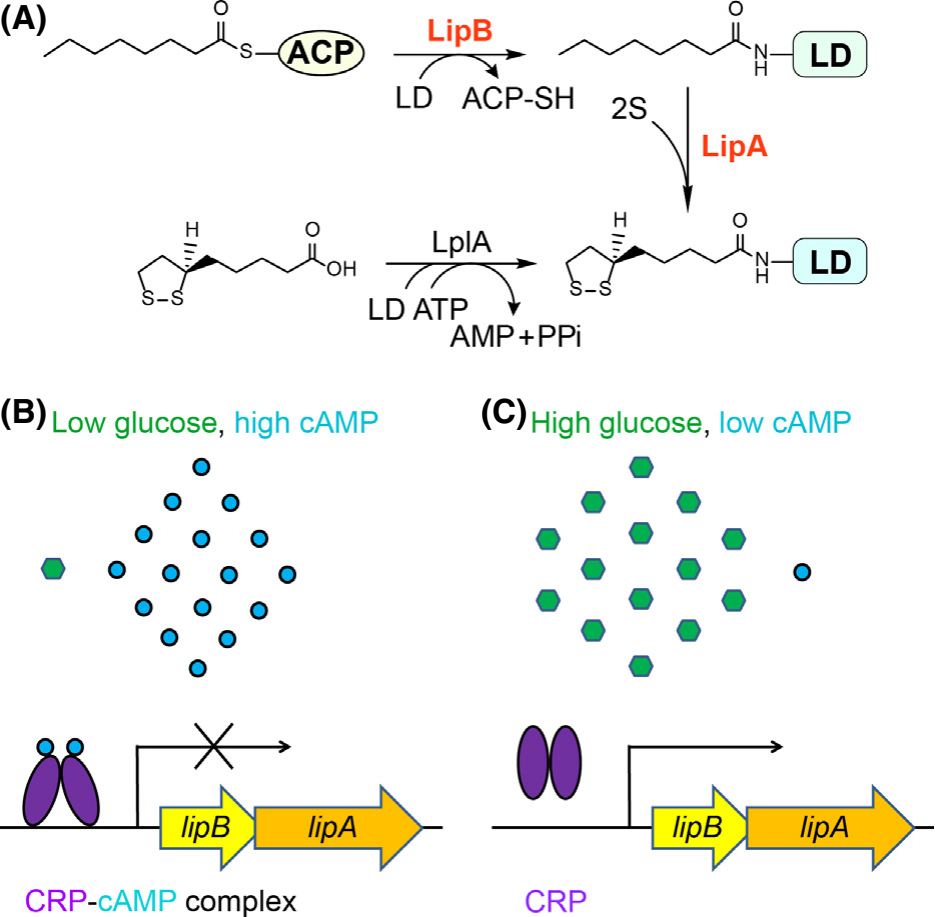
Working model for pathway for bacterial lipoic acid metabolism and its possible regulation. (A) A pathway proposed for lipoic acid synthesis and its scavenging in *Shewanella*. LipA, lipoic acid synthase; LipB, Octanoyl-ACP: protein ligase (*N*-octanoyltransferase); PdhR, pyruvate dehydrogenase operon repressor; LD, lipoyl domain (in light blue); ACP, acyl carrier protein (in white oval). *Shewanella lipB/A* expression is shut off by the cAMP-CRP complex on the condition of low glucose level (or high cAMP concentration) (B), whereas is induced upon high concentration of glucose is available (or cytosolic cAMP is limited) (C). Blue dots denote cAMP molecules, green regular hexagon represents glucose, and purple ovals indicate CRP protein. CRP, cAMP-receptor protein.

Most of current knowledge of lipoic acid metabolisms comes from studies with *E. coli* (Zhao et al. 2003; Cronan et al. 2005). Two alternative strategies have been developed in *E. coli* to satisfy the trace physiological demand for lipoic acids. It includes de novo biosynthesis pathway and the scavenging route (Cronan et al. 2005; Hermes and Cronan 2009; Rock 2009; Christensen and Cronan 2010). The former pathway is constituted of two consecutive steps: the LipB (octanoyl-ACP: protein *N*-octanoyl-transferase) transfers the endogenously produced octanoyl moieties from octanoyl-ACP (an intermediate of the fatty acid biosynthesis) to lipoyl domains (Fig.1A) (Jordan and Cronan 2003; Zhao et al. 2003, 2005); in the second step the LipA (lipoyl synthase) uses S-adenosyl-l-methionine (SAM)-dependent radical chemistry to insert two sulfur atoms at carbons 6 and (Fig.1A) (Zhao et al. 2003; Cronan et al. 2005; Douglas et al. 2006; Christensen and Cronan 2010). The lipoyl protein ligase (LplA) plays a critical role in utilization of exogenous lipoic acids from environments in which the lipoyl-adenylate intermediate is required (Fig.1A) (Morris et al. 1994, 1995; Reed et al. 1994). Although the metabolic mechanism of lipoic aids that was initially discovered in the early of 1940s (Reed 2001) was extensively investigated (Reed 2001; Cronan et al. 2005; Hermes and Cronan 2009; Rock 2009; Christensen and Cronan 2010), its genetic regulation/control is poorly understood (Kaleta et al. 2010; Feng and Cronan 2014).

cAMP receptor protein (CRP, also called catabolic activator protein, CAP) is a type of global regulator, representing a classical model for bacterial gene regulation systems(Green et al. 2014). The paradigm version of CRP is *E. coli crp* protein product that modulates expression of hundreds of genes involved in a variety of bacterial physiological aspects such as energy metabolism (e.g., galactose catabolism) (Zheng et al. 2004; Green et al. 2014). Indeed, the activity of CRP requires the presence of its physiological ligand/effector, cyclic AMP (cAMP) (Zheng et al. 2004; Green et al. 2014). Upon the CRP protein is occupied by the cAMP small molecule, it proceeds to an allosteric alteration/structural rearrangement, allowing its acquisition of an ability to specifically bind a collection of specific target DNA sequences(Schultz et al. 1991; Green et al. 2014). As we know, the typical CRP box (cAMP-CRP binding site) is referred to the imperfect palindromic consensus sequence “N_3_TGTGAN_6_TCACAN_3_” (Zheng et al. 2004). In the similarity to the well-studied FadR regulator that has dual functions in fatty acid metabolism (Feng and Cronan 2009a,b, 2010, 2012), it appears that the dimeric CRP protein-mediated regulation also can exert two opposite roles, i.e., either activation (Hanamura and Aiba 1992; Ishizuka et al. 1994; Zheng et al. 2004) or repression (Aiba 1983; Hanamura and Aiba 1991; Ishizuka et al. 1994) in response to distinct external and/or internal stimuli/inputs (Green et al. 2014). Recently, comparative genomics-based reconstruction of bacterial regulatory networks RegPrecise (http://regprecise.lbl.gov/RegPrecise) by Rodionov's research group (Rodionov et al. 2011; Novichkov et al. 2013) predicted that a possible CRP box (AAGTGTGATCTATCTTACATTT) is present in front of *lipBA* operon (SO1162-S01161) of *Shewanella oneidensis* MR-1(a marine-borne species of *γ*-proteobacteria family) with considerable potential for the remediation of contaminated environments and application in microbial fuel cells (Fredrickson and Romine 2005; Fredrickson et al. 2008).

More importantly, it seemed likely that the predicted site reflects an evolutionally conserved regulatory mechanism in that it is found in nearly all the *Shewanella* species with known genome sequences and similar scenario were seen even with the two human pathogens *Salmonella typhimurium* and *Klebsiella pneumonia*. This might raise a possibility that cAMP signaling is linked to bacterial lipoic acid synthesis in certain species of *γ-*proteobacteria. However, this hypothesis requires further *in vitro* and *in vivo* experimental verification.

In this paper, we aimed to resolve this unanswered question. As anticipated, electrophoresis mobility shift assays (EMSA), we conducted and confirmed that the two CRP proteins of *E. coli* and *Shewanella* are functionally exchangeable and the predicted CRP sites of *Shewanella* are functional. Using the chromosome *lipBA-lacZ* transcriptional fusion in *E. coli*, we visualize that the removal of *E. coli crp* gene gave above fourfold increment of *lipBA* promoter-driven *β*-gal expression, which is almost identical to the scenario seen with *Shewanella*. Somewhat it is unexpected, but not without precedent that an addition of glucose into media effectively induces *lipBA* expression in *Shewanella*, in that the lowered cAMP level relieves the repression of *lipBA* by cAMP-CRP complex (Fig.1B and C). Therefore, our finding answered the long-term unresolved question in the field of lipoic acid metabolism and might represent a first paradigm illustrating the genetic control of bacterial lipoic acid synthesis by cAMP-dependent CRP signaling in certain species of *γ*-proteobacteria.

## Materials and Methods

### Bacterial strains and growth conditions

The bacterial strains used here were derivatives of both *E. coli* K-12 and *S. oneidensis* MR-1 (Table1) and cultivated aerobically at 37°C and 30°C, respectively. For the growth of *E. coli*, the following three media were utilized, including Luria-Bertani (LB) medium (10 g of tryptone, 5 g of yeast extract and 10 g of NaCl per liter; pH 7.5), rich broth (RB) medium (10 g of tryptone, 1 g of yeast extract, and 5 g of NaCl per liter), and M9 minimal medium with either 5 mmol/L sodium acetate or 0.4% glucose as the sole carbon source (Feng and Cronan 2009b, 2010). M1-defined medium containing 0.02% (w/v) of vitamin-free casamino acids and 15 mmol/L lactate as electron donor was used to cultivate *S. oneidensis* (Gao et al. 2008). If required, chemicals or antibiotics were added as follows: 2,6-diaminopimelic acid (DAP), 0.3 mmol/L; sodium ampicillin, 100 *μ*g/mL; kanamycin sulfate, 25 *μ*g/mL; and tetracycline, 15 *μ*g/mL; gentamycin, 15 *μ*g/mL.

**Table 1:** Bacterial strains and plasmids in this study

Bacteria or plasmids	Relevant characteristics	Refs or origins
Bacterial strains		
*Escherichia coli*		
BL21(DE3)	Engineered *E. coli* strain as an expression host for recombinant plasmids	Lab stock
MG1655	Wild type of *E. coli* K-12 (F-, *λ*^*-*^, *rph-1*)	CGSC^1^, Lab stock
WM3064	Donor strain for conjugation; Δ*dapA*	W. Metcalf, UIUC
BW25113	A Δ*lac* strain of *E. coli* K-12 (F-, *λ*^*-*^, *rph-1*, Δ(*araD-araB*)*567* Δ*lacZ4787*(::*rrnB*-3) Δ(*rhaD-rhaB*)*568* *hsdR514*)	CGSC^1^, Baba et al. (2006)
JW5702-4	(BW25113, Δ*crp*-765*::*kan)	CGSC^1^, Baba et al. (2006), Feng and Cronan (2010)
MC1061	F-, *λ*^-^, Δ(*araA-leu*)7697, [*araD139*]_B/r_, Δ(*codB-lacI*)*3*, *galK16*, *galE15*(*GalS*), *e14*^-^, *mcrA0*, *relA1*, *rpsL150*(strR), *spoT1*, *mcrB1*, *hsdR2*	Lab stock, Casadaban and Cohen (1980), Feng and Cronan (2009b)
RH77	MC4100, Δ*cyaA*, Δ*crp::*Tn*10*	Lab stock Feng and Cronan (2010, 2012)
DH5*α* (*λ-pir*)	An *E. coli* Δ*lac* host for pAH125 and its derivatives	Feng and Cronan (2009a, 2012), Haldimann and Wanner (2001)
FYJ208	*Vibrio cholerae* O395	Jame Jun Zhu's lab
FYJ239	BL21(DE3) carrying pET28-*crp*_ec	Feng and Cronan (2012), Goble et al. (2013)
FYJ426	*Salmonella enterica* serovar Typhimurium 14028s	Slauch's lab
FYJ452	DH5a(*λ*-*pir*) carrying pAH-P*lipBA*_she	This work
FYJ453	MC4100 whose chromosome was integrated with the *lipBA*_she*-lacZ* transcriptional fusion at the *λ* phage site	This work
FYJ457	MC1061, *lipBA*_she*-lacZ* transcriptional fusion	P1_*vir*_(FYJ453) × MC1061^2^, This work
FYJ458	MC4100, Δ*cyaA*, Δ*crp::*Tn*10*, *lipBA*_she*-lacZ* transcriptional fusion	P1_*vir*_(FYJ453) × RH77^2^, This work
FYJ462	Topo carrying pET28-*crp*_she	This work
FYJ463	BL21(tuner) carrying pET28-*crp*_she	This work
*S. oneidensis*		
MR-1	Wild-type	Gao's lab
HG0624	Δ*crp* derived from MR-1	Gao et al. (2010)
HG1162-1	Δ*lipBA* derived from MR-1	This work
HG0424	Δ*aceE* derived from MR-1	This work
HG1329	Δ*cyaC* derived from MR-1	This work
Plasmids		
pET28(a)	Commercial T7-driven expression vector, Km^R^	Novagen
pET28-*crp*_ec	pET28(a) carrying *E. coli crp* gene, Km^R^	Feng and Cronan (2012), Goble et al. (2013)
pAH125	A promoter-less *lacZ* reporter plasmid in *E. coli*, Km^R^	Haldimann and Wanner (2001)
pHG101	A promoter-less broad-host Km^R^ vector	Wu et al. (2011)
pHGEI01	An integrative *lacZ* reporter vector	Fu et al. (2014)
pAH-P*lipBA*_she	A pAH125 derivative encoding *Shewanella oneidensis lipBA* promoter region (∽350 bp)	This work
pET28-*crp*_she	pET28(a) encoding *S. oneidensis crp* gene, Km^R^	This work

1 CGSC denotes Coli Genetic Stock Center, Yale University.

2 Selection for kanamycin resistance.

### Plasmids and DNA manipulations

Using polymersase chain reaction (PCR) with primers *crp*_she-F plus *crp*_she-R (Table2), the *S. oneidensis crp* gene was amplified and inserted into the BamHI and XhoI sites of pET28a(+) expression vector, giving the recombinant plasmid pET28-*crp*_she (Table1). The promoter region of *S. oneidensis lipBA* (referred to P*lipBA*_she) covering the predicted CRP site (Table3) was amplified with a set of specific primers *PlipBA*-F plus *PlipBA*-R and cloned into the two cuts SalI and EcoRI of promoter-less plasmid pAH125 to give the recombinant plasmid pAH-P*lipBA*_she. Consequently, the pAH-P*lipBA*_she plasmid was transformed into MC4100 (Δ*lac*), resulting in the LacZ reporter strain FYJ453 with P*lipBA*_she-lacZ transcriptional fusion on chromosome (Table1). The inserts introduced in the recombinant plasmids we generated were validated by both PCR assays and direct DNA sequencing (Feng and Cronan 2011a,b).

**Table 2:** DNA oligonucleotide sequences used in this work

Primers	Primer sequences	Purposes
*crp*_she-F (BamHI)	5′-CG *GGATCC* ATG GCT CTG ATT GGT AAG CC-3′	Gene cloning
*crp*_she-R (XhoI)	5′-CCG *CTCGAG* TTA ACG GGT ACC ATA TAC CAC-3′	
*crp*_she-ck1	5′-GTG AAT CCA GTG AGT TTG ACA-3′	PCR detection for the *crp* mutant of *Shewanella*
*crp*_she-ck2	5′-CAG AGT TGA CTA ACG CCT TG-3′	
P*lipBA*-F (SalI)	5′-CCG *GTCGAC* GAT GAA CTG ATG GAG TTC CCC-3′	PCR amplification and cloning of the *lipBA* promoter
P*lipBA*-R (EcoRI)	5′-AACC *GAATTC* CAA GGG CAA CCT CTC CCC TA-3′	
*lipBA_she* CRP site-F (43 bp)	5′-CAA GGT CAT A**AA GTG TGA TCT ATC TTACATTT**A TGG CCA AGA G-3′	Synthesis of the predicted CRP site of *Shewanella lipBA*
*lipBA_she* CRP site-R (43 bp)	5′-CTC TTG GCC AT**A AATGTA AGA TAG ATC ACACTT** TAT GAC CTT G-3′	
*lipA_ec* CRP site-F (42 bp)	5′-ACG GAG TAA T**AGATG TTA TCC GTA ATG CATTT**T GAA AAA GTA-3′	Synthesis of the suspected CRP site of *E. coli lipA*
*lipA_ec* CRP site-R (42 bp)	5′-TAC TTT TTC A**AA ATG CAT TAC GGA TAA CAT CT**A TTA CTC CGT-3′	
*fadD*_ec CRP site-F (48 bp)	5′-GTA AAG ATA AAA ATA **AAT AGT GAC GCG CTTCGC AACC**TT TTC GTT GGG-3′	Synthesis of the known CRP site of *E. coli fadD*
*fadD*_ec CRP site-R (48 bp)	5′-CCC AAC GAA AA**G GTT GCG AAG CGC GTC ACTATT** TAT TTT TAT CTT TAC-3′	
*ybeD*_ec CRP site-F(42 bp)	5′-AAA CAC TTG A**AAGTG TAA TTT CCG TCC CCATA**T ACT AAG CAT-3′	Synthesis of the anticipated CRP site of *E. coli ybeD*
*ybeD*_ec CRP site-R(42 bp)	5′-ATG CTT AGT A**TATGG GGA CGG AAA TTA CACTT**T CAA GTG TTT-3′	
*ybeD*_es CRP site-F(42 bp)	5′-GAA CAC TTG A**AA GTG TGA TTT CCA TCC CCA TA**T ACT AGG TAT-3′	Synthesis of the CRP site of *ybeD* gene from *Enterobacter* sp. 638
*ybeD*_es CRP site-R(42 bp)	5′-ATA CCT AGT **ATA TGG GGA TGG AAA TCA CAC TT**T CAA GTG TTC-3′	
*ybeD*_kp CRP site-F(42 bp)	5′-GAACACTTGA**AA GTG TGA TTT CCA TCC CCA TA**TACTATTCAT-3′	Synthesis of the CRP site of *ybeD* gene from *Klebsiella pneumonia*
*ybeD*_kp CRP site-R(42 bp)	5′-ATG AAT AGT A**TATGG GGA TGG AAA TCA CACTT**T CAA GTG TTC-3′	
*ybeD*_st CRP site1-F(42 bp)	5′-GAA CGCTTGA**AA GTG TGA TTT TCG TCC CCA TA**T ACTATGCAT-3′	Synthesis of the CRP site 1 of *ybeD* gene from *Salmonella typhimurium* LT2
*ybeD*_st CRP site1-R(42 bp)	5′-ATG CAT AGT A**TA TGG GGA CGA AAA TCA CACTT**T CAA GCG TTC-3′	
*ybeD*_st CRP site2-F(42 bp)	5′-CTG TGG CGG G**AG TTG TTA TTT TTT TTA CGT AA**T GCC GGA GCT-3′	Synthesis of the CRP site 2 of the *ybeD* gene from *Salmonella typhimurium* LT2
*ybeD*_st CRP site2-R(42 bp)	5′-AGC TCC GGC A**TTACG TAA AAA AAA TAACAACT**C CCG CCA CAG-3′	
*ybeD*_yp CRP site-F (42 bp)	5′-ATT GGC CCC A**TATTG TGA TTA ATC TTA TAT TG**C AAA TAA GCT-3′	Synthesis of the CRP site of the *ybeD* gene from *Yersinia pestis*
*ybeD*_yp CRP site-R (42 bp)	5′-AGC TTA TTT G**CA ATA TAA GAT TAA TCA CAA TA**T GGG GCC AAT-3′	
LacZ-R	5′-CAG TGA ATC CGT AAT CAT GGT C-3′	PCR assay for the *lipBA-lacZ* junction
*crp_ec*-F	5′-CAG GTA GCG GGA AGC ATA TTT C-3′	PCR assay for the *E. coli crp*
*crp_ec*-R	5′-CAG CGT TTG TCG AAG TGC ATA G-3′	
*ybeD*-F (19-39)	5′-GAT GAA CTG ATG GAG TTC CCC-3′	PCR (RT-PCR) assay for the *S. oneidensis ybeD*
*ybeD*-R (223-243)	5′-GAT GTT GGC GAG CTC TGT GTA-3′	
*lipB*-F (471-491)	5′-CTG TGG ATC GTT GAA CAT CCA-3′	PCR (RT-PCR) assay for the *S. oneidensis lipB*
*lipB*-R (760-780)	5′-GAC CTA AGG AAG CCA CTT TGC-3′	
*lipA*-F (1020-1040)	5′-CTG AAC GTT TAC AAC CCG GAG-3′	PCR (RT-PCR) assay for the *S. oneidensis lipA*
*lipA*-R (1253-1273)	5′-CAT AAA GGT TGC TGT GCC GTG-3′	
*ybeD-lipB*-F (208-229)	5′-CAT ATC GAA ACC CTG TAC ACA G-3′	PCR (RT-PCR) assay for the *ybeD-lipB* junction of *S. oneidensis*
*ybeD-lipB*-R (471-492)	5′-GTG GAT GTT CAA CGA TCC ACA G-3′	
*lipB-lipA*-F (910-930)	5′-GCC CAC AAA CTG TGA TAG AAG-3′	PCR (RT-PCR) assay for the *lipA-lipB* junction of *S. oneidensis*
*lipB-lipA*-R (1160-1181)	5′-CTT GCT TAA TGT CGA GAA TGC G-3′	
*lipBA*-Nest (769-789)	5′-GGA TCC TAA GAC CTA AGG AAG-3′	5′-RACE of *S. neidensis lipBA*
*lipBA*-GSP (868-898)	5′-CTG CAT TGC ACC ATT TCA AGG-3′	
*16S_*she-F	5′-GAT AAC AGT TGG AAA CGA CTG-3′	PCR (RT-PCR) assay
*16S*_she-R	5′-CTT TCC TCC CTA CTG AAA GTG-3′	

The underlined italic letters represent restriction sites, and the bold letters denote the known (and/or predicted) CRP-binding sites. RT-PCR, reverse transcription-polymersase chain reaction; CRP, cAMP-receptor protein.

**Table 3:** CRP binding sites in front of potential *lipB/A* operons from a variety of species amongst *γ*-proteobacteria

Organisms	Gene	Loci	CRP site	Position^1^	Score
*Enterobacter* sp. 638	*ybeD*	Ent638_1166	AAGTGTGATTTCCATCCCCATA	−90	4.4
*Escherichia coli* MG1655	*ybeD*	b0631	AAGTGTAATTTCCGTCCCCATA	−94	3.6
*Cistrobacter koseri*	*ybeD*	CKO_02527	AAGTGTGATTTCCATCCCCATA	−91	4.4
*Klebsiella pneumonia*	*ybeD*	KPN_00663	AAGTGTGATTTCCATCCCCATA	−97	4.4
*Salmonella typhimurium* LT2	*ybeD*	STM0636	AGTTGTTATTTTTTTTACGTAA AAGTGTGATTTTCGTCCCCATA	−35 −93	3.9 4.2
*Yersinia pestis*	*ybeD*	y1174	TATTGTGATTAATCTTATATTG	−146	4.2
*Shewanella baltica*	*lipB*	Sba_3281	AAATGTGATCTGTCTTACATTT	−74	5.2
*S. halifaxensis*	*lipB*	ShaI_3240	AAATGTGATCCGTATTACATTT	−76	5.2
*S. loihica*	*lipB*	Shew_2941	AAATGTGATCTACCTTACATTT	−70	5.3
*S. oneidensis*	*lipB*	SO1162	AAGTGTGATCTATCTTACATTT	−68	5.1
*S. pealeana*	*lipB*	Spea_3155	AAATGTGATCCGTATTACATTT	−76	5.2
*S. piezotolerans*	*lipB*	swp_3928	AAATGTGATCTGTCTTACATTT	−69	5.2
*S. putrefaciens*	*lipB*	Sputcn32_2875	AAATGTGATCTATCTTACATTT	−69	5.3
*S. sediminis*	*lipB*	Ssed_3491	AAATGTGATCTAGCTTACATTT	−75	5.3
*S. woodyi*	*lipB*	swoo_3714	AAGTGTGATCTAGCTTACAATT	−74	5.1
*S*. sp ANA-3	*lipB*	Shewanan3_0989	AAATGTGATCTGTCTTACATTT	−74	5.2
*S*. sp MR-4	*lipB*	Shewmr4_0985	AAATGTGATCTGTCTTACATTT	−74	5.2
*S*. sp MR-7	*lipB*	Shewmr7_1050	AAATGTGATCTGTCTTACATTT	−74	5.2
*S*. sp W3-18-1	*lipB*	Sputw3181_1028	AAATGTGATCTATCTTACATTT	−75	5.3

CRP, cAMP-receptor protein.

1 The position is relative to the translation initiation site. All the information is sampled from the RegPrecise database (http://regprecise.lbl.gov/RegPrecise/search.jsp).

The *lipBA* promoter activity was assessed using an integrative *lacZ* reporter system as described recently (Fu et al. 2014). A fragment covering the sequence upstream of the *lipB* gene from −300 to +1 was amplified and cloned into the reporter vector pHGEI01, verified by sequencing, and the correct plasmid was then transferred into *S. oneidensis* strains by conjugation. Proper integration of the promoter fusion constructs was confirmed by PCR. To eliminate the antibiotic marker, the helper plasmid pBBR-Cre was transferred into the strains carrying the correct integrated construct. Colonies without the integrated antibiotic marker were screened and verified by PCR, and followed by the loss of pBBR-Cre as described previously (Fu et al. 2013).

### In-frame mutant construction and complementation

In-frame deletion strains for *S. oneidensis* were constructed using the *att*-based Fusion PCR method as described previously (Jin et al. 2013). In brief, two fragments flanking gene of interest were amplified by PCR, which were linked together by a second round of PCR. The fusion fragments were introduced into plasmid pHGM1.0 by using Gateway BP clonase II enzyme mix (Invitrogen, Grand Island, NY, USA) according to the manufacturer's instruction, resulting in mutagenesis vectors in *E. coli* WM3064, which were subsequently transferred into *S. oneidensis* MR-1 via conjugation. Integration of the mutational constructs into the chromosome was selected by resistance to gentamycin and confirmed by PCR. The verified transconjugants were grown in LB broth in the absence of NaCl and plated on LB supplemented with 10% sucrose. Gentamycin-sensitive and sucrose-resistant colonies were screened by PCR for deletion of the target gene. Mutants were verified by direct sequencing of the mutated regions.

Plasmids pHG101 and pHG102 were used in genetic complementation of mutants (Wu et al. 2011). For complementation of genes next to their promoter, a fragment containing the gene of interest and its native promoter was generated by PCR and cloned into pHG101. For the remaining genes, the gene of interest was amplified and inserted into MCS of pHG102 under the control of the *arcA* promoter, which is constitutively active (Gao et al. 2010). The resulting vectors were transferred into its corresponding mutant strain via conjugation and its presence was confirmed by plasmid purification and restriction enzyme digestion.

### P1_*vir*_ phage transductions

Following the protocol described by Miller (1992), we conducted the experiment of P1_*vir*_ transduction. Transduction of strain MC1061 with a lysate grown on FYJ453 (P*lipBA_she-lacZ*) with selection for kanamycin resistance gave strain FYJ457 (MC1061, P*lipBA_she-lacZ*). Strain FYJ458 was constructed by transduction of strain RH77 (MC4100, Δ*crp::*Tn*10*) with a P1_*vir*_ lysate grown on FYJ457 (MC1061, P*lipBA_she-lacZ*) with selection for kanamycin resistance (Table1). All the relevant genotypes were determined using PCR with a primer set (e.g., P*lipBA*-F plus LacZ-R, Table2), and the PCR products were confirmed by direct DNA sequencing (Feng and Cronan 2012).

### RNA isolation and RT-PCR

Mid-log phase cultures of *S. oneidensis* MR-1 grown in RB media were collected for total bacterial RNA preparations. As we did before, the RNeasy bacterial RNA isolation kit (Qiagen, Hilden, Germany) was adopted (Feng and Cronan 2009b; Feng et al. 2013b). The quality of the acquired RNA samples was visualized using agarose gel electrophoresis. Using the general PCR assay in which the total RNA samples function as templates with primers *16S_*she-F plus *16S*_she-R (Table2), the possible contamination of trace genomic DNA in the RNA samples was routinely figured out as we described earlier (Feng and Cronan 2009b, 2010).

On the basis of above qualified RNA samples, we performed the reverse transcription (RT)-PCR experiments (Feng and Cronan 2009b, 2010). Briefly, 1 *μ*g of total RNA was mixed with 0.5 *μ*g of random primers (11 *μ*L in total), denatured (70°C for 5 min), and then chilled on ice (5 min). The RT reaction mixture (20 *μ*L total volume) comprised 10 *μ*L of denatured RNA template, 1 *μ*L of random primers, 4 *μ*L of ImProm-II 5× reaction buffer, 2.5 *μ*L of 1 mol/L MgCl2, 1 *μ*L of deoxynucleoside triphosphate mix, 0.5 *μ*L of the recombinant RNasin RNase inhibitor, and1 *μ*L of ImProm-II reverse transcriptase (Feng and Cronan 2009b, 2011a). The program for RT reaction included the equilibration at 25°C for 5 min, an extension at 42°C for 60 min, and the inactivation of enzyme at 70°C for 15 min. As a result, the cDNA pool (1 *μ*L) was used as the template to PCR-amplify the *lipBA* operon-related genes/DNA fragments.

### Real-time quantitative RT-PCR

On the basis of SYBR Green dye method as we previously mentioned (Feng and Cronan 2009b, 2010), real-time quantitative RT-PCR (qRT-PCR) experiments were employed to evaluate the altered expression profile of *S. oneidensis lipBA* operon in the Δ*crp* mutant. qPCR reaction system (20 *μ*L) contained 12.5 *μ*L of iQ™ SYBR Green Supermix, 1 *μ*L of each primer, 1 *μ*L of the diluted cDNA sample, and 4.5 *μ*L of sterile water. All the data were collected in triplicate on a Mastercycler® eprealplex (Eppendorf, Hauppauge, NY, USA), using the program of a denaturing cycle at 95°C for 15 min, 45 cycles comprising 94°C for 20 sec, 60°C for 20 sec, and 72°C for 20 sec, and a final step featuring with gradient temperature from 60°C to 90°C for dissociating double stranded DNA products. The reference gene was the16*S*_she rRNA-encoding gene (Table2) and water acted as blank control to monitor cross-contamination of various cDNA samples. The relative expression levels were calculated with the 2^−ΔΔ*C*^_T_ method developed by Livak and Schmittgen (2001).

### 5′-RACE

RLM-RACE (Ambicon, Grand Island, NY, USA), an improved version of 5′-RACE kit, was applied in mapping the transcription start site of *S. oneidensis lipBA* operon (Feng and Cronan 2011a,b). The nested PCR reactions were established using two sets of combined primers (Outer Primer plus *lipBA*-GSP and Inner Primer plus *lipBA*-Nest primer) (Table2). The PCR program was described with a denaturing cycle at 95°C for 5 min followed by 35 cycles comprising 95°C for 30 sec, 55°C for 30 sec, and 72°C for 30 sec. The purified PCR products were sent for direct DNA sequencing. The transcriptional start site was assigned to first nucleotide adjacent to the RLM-RACE adaptor (Feng and Cronan 2009a,b; Feng et al. 2013b).

### Enzymatic assays

For PDH assay, cells were grown at 30°C in 25 mL of LB containing the appropriate antibiotics until the beginning of the stationary phase, harvested, and washed twice with a 0.04 mol/L potassium phosphate buffer (pH 7.5). The resulting pellets were frozen rapidly and stored at −80°C. Cell extracts were prepared by resuspending the thawed pellets in 2 mL of the same buffer prior to sonication with a microtip in a Branson model 200 Sonifier (2 min total, with 40-sec pulses at 20-sec intervals). Cell debris was removed by centrifugation (10 min at 12,000*g* and 4°C), and the supernatants were used for assays at 25°C as described previously (Reed and Cronan 1993). Protein concentrations were determined by the bicinchoninic acid protein assay reagent (Pierce Chemical Co., Rockford, IL, USA).

To measure the *β*-galactosidase activities in *E. coli*, bacterial lysates from mid-log phase cultures grown in LB (or M9) media were prepared by treatment with sodium dodecyl sulfate-chloroform (Miller 1972; Feng and Cronan 2009b). Similarly, cells of *S. oneidensis* (mid-log phase under experimental settings) were pelleted for assaying its *β*-galactosidase activity with an assay kit as described previously (Wu et al. 2011).

### Measurement of intracellular cAMP levels

Cells in mid-log phase cultures (∽0.3 of OD_600_) were collected by centrifugation and washed twice with charcoal-treated phosphate-buffered saline (PBS; pH 7.0). Both supernant and pellet fractions were applied to the cAMP assay using Cyclic AMP EIA kit (Cayman Chemical Co., Ann Arbor, Michigan, USA) according to the manufacturer's instruction.

### Expression, purification and identification of two CRP proteins

To prepare the recombinant CRP protein in two versions (CRP_ec and CRP_she), the engineered *E. coli* strains carrying either pET28-*crp_*ec or pET28-*crp_*she (Table1) were induced with 0.3 mmol/L isopropyl *β*-d-1-thiogalactopyranoside (IPTG) at 30°C for 5 h(Feng and Cronan 2012). Following bacterial lysis by a French pressure cell, the clarified supernatants by centrifugation (30,966*g*, 30 min) were loaded onto a nickel chelate column (Qiagen). After removal of the contaminated protein by washing with 1×phosphate buffered saline (PBS) with 50 mmol/L imidazole, the interested CRP proteins (CRP_ec or CRP_she) were eluted using elution buffer containing 150 mmol/L imidazole. Finally, the protein was concentrated by ultrafiltration (30 kDa cut-off) and exchanged into 1× PBS (pH 7.4) containing 10% glycerol. The purity of the recombinant CRP proteins was judged by 12% sodiumdodecyl sulphate-polyacrylamide gel electrophoresis (SDS-PAGE) (Feng and Cronan 2009b, 2011b). To verify the identity of the acquired proteins, the de-stained (SDS-PAGE) gel slices were subjected to liquid chromatography quadrupole time-of-flight mass spectrometry using a Waters Q-Tof API-US Quad-ToF mass spectrometer linked to a Waters nanoAcquity UPLC(Feng and Cronan 2011a; Feng et al. 2013a,b).

### Electrophoretic mobility shift assays

The function of the predicted CRP-binding site of *Shewanella lipBA* operon was assessed in vitro using electrophoretic mobility shift assays (EMSA) with little improvements (Feng and Cronan 2011a; Goble et al. 2013; Feng et al. 2014). In the EMSA tests, nine pieces of DNA probes were composed of seven suspected probes (*lipBA*_she, *ybeD*_ec, *ybeD*_es, *ybeD*_kp, *ybeD*_st1, *ybeD*_st2, and *ybeD*_yp) and the two control probes, the *fadD*_ec site with known function (the positive control) and the *lipA*_ec without any function (the negative control) (Table3). The digoxigenin (DIG)-labeled DNA probes were prepared in vitro through annealing two complementary oligonucleotides in TEN buffer (10 mmol/L Tris-HCl, 1 mmol/L EDTA, 100 mmol/L NaCl; pH 8.0) and then labeled by the terminal transferase with DIG-ddUTP (Roche, Indianapolis, IN, USA) (Feng et al. 2014).

In the presence/absence of cAMP (20 pmol), the various DIG-labeled DNA probes (0.2 pmol) were incubated with or without CRP protein in the binding buffer (Roche) at room temperature for around 20 min. Following the separation of the DNA-protein complexes with a native 7% PAGE gel, the chemiluminescent signals were further captured by the exposure to ECL film (GE Healthcare, Piscataway, NJ, USA) (Feng and Cronan 2011b, 2012).

### Bioinformatic analyses

The alignments of DNA (and/or protein) sequences were conducted using the ClustalW2 program (http://www.ebi.ac.uk/Tools/clustalw2/index.html) and final output was processed by the ESPript 2.2 server (http://espript.ibcp.fr/ESPript/cgi-bin/ESPript.cgi). The *lipBA* regulons and the possible CRP-recognizable sites of *γ*-proteobacteria were collected from the RegPrecise database (Novichkov et al. 2010a) and were analyzed (Feng et al. 2013a) using RegPredict software (Novichkov et al. 2010b). The sequence logo for the CRP consensus palindrome was generated by WebLogo (http://weblogo.berkeley.edu/logo.cgi). The software of SPDBV_4.01 (http://spdbv.vital-it.ch/) was used for structure modeling.

## Results

### *Shewanella lipBA* is an operon

**Figure 2:**
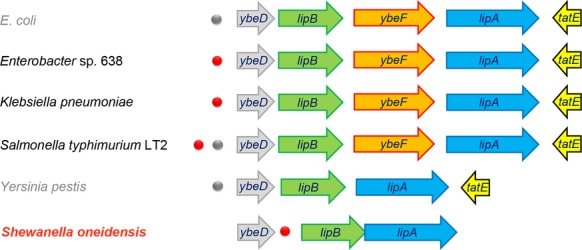
Gemomic context of the *lipB*/*A* operon/genes in the selected *γ*-proteobacteria. Blue arrows represent the *lipA* genes that encode the lipoic acid synthase catalyzing the last committed reaction of lipoic acid biosynthesis pathway, whereas green arrows indicate the octanoyl-protein ligase-encoding genes (*lipB*).The gray arrow upstream of the *lipB* gene refers to the *ybeD* gene of unknown function. In some cases, the *tatE* gene (Sec-independent protein translocase) downstream of *lipA* is shown with yellow arrow. In the four species (*Escherichia coli*, *Enterobacter* sp. 638, *Klebsiella pneumoniae*, and *Salmonella typhimurium* LT2), the *ybeF* gene encoding an LysR-type transcription factor (in orange) is located between *lipA* and *lipB*. The predicted CRP-binding palindromes are highlighted with dots (red dots represent the experimentally verified sites, whereas the gray ones are not experimentally validated). CRP, cAMP-receptor protein.

The paradigm pathway of lipoic acid synthesis is encoded by two genes *lipB* and *lipA* of *E. coli*. The two sequential steps of this pathway included LipB-catalyzed transfer of octanoyl moiety from octanoyl-ACP to lipoyl domains of the cognate enzymes and LipA-mediated insertion of sulfur atoms at C6 and C8 of LD-bound octanoyl moiety to give lipoate (Fig.1). Therefore, we are interested in examining the genetic context of the *lipB* and/or *lipA* in *γ*-proteobacteria using RegPredict software (Novichkov et al. 2010b) (Fig.2). In addition to the reference strains (e.g., *E. coli*, *Salmonella enterica*, *Yersinia pestitis*, etc.), all the other samples are focused on *Shewanella* species from the RegPrecise database (Novichkov et al. 2010a). We noted that the *ybeD* (*SO1163*) gene is constantly present upstream of the *lipB* gene (Fig.2), and YbeD protein of *E. coli* origin exhibits a striking structural homology to the allosteric regulatory domain of d-3-phosphoglycerate dehydrogenase (Kozlov et al. 2004).

**Figure 3:**
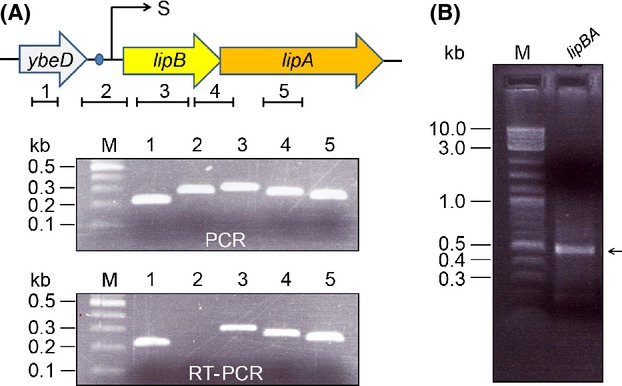
Determination of the *Shewanella lipBA* as an operon. (A) Genetic organization and transcriptional analyses of *Shewanella lipBA* operon. The three arrows represent *ybeD* (in gray), *lipB* (in yellow) and *lipA* (in orange), respectively. The numbered short lines (1, 2, 3, 4, and 5) indicate the specific PCR amplicons. The transcription start sites (S) is indicated with an arrow. The PCR and RT-PCR products were separated by the electrophoresis of 1.5% agarose gel. (B) Electrophoretic analyses for the 5′-RACE product of *Shewanella lipBA* operon 5′-RACE product were separated with 2.0% agarose gel and the expected size was highlighted with an arrow. kb, kilo-base pair; M, 100 bp DNA ladder (New England Bio-labs, Ipswich, MA, USA). RT-PCR, reverse transcription-polymersase chain reaction.

Unlike the scenario seen with *E. coli* that *lipB* and *lipA* are separated by a gene (*ybeF*) encoding a LysR-family transcription factor of unknown function (Feng and Cronan 2014) (Fig.2), it seemed likely that *lipB* and *lipA* constitutes an operon in most of species of *Shewanella* (Fig.1). Although physiological advantages for the co-transcription of these two genes are expected, experimental evidence is lacking. To address this hypothesis, the strain of *S. oneidensis* MR-1 was selected for our experiments. We established the combined PCR and RT-PCR assays using five pairs of specific primer pairs (Table2 and Fig.3A). The positive amplifications (1, 3 and 5) were obtained by both PCR and RT-PCR showed that all three genes (*ybeD*, *lipB* and *lipA*) are transcribed (Fig.3A). The fact that the primed amplicon (designated to 2) was observed only by PCR, but not by RT-PCR suggested that *ybeD* is not co-transcribed together with *lipB* (Fig.3A). As anticipated, the designed amplicon covering both *lipB* and *lipA* was positive in both PCR and RT-PCR assays, validating that *lipB* and *lipA* act as an operon (transcriptional unit) (Fig.3A).

### *S. oneidensis lipBA* promoter

**Figure 4:**
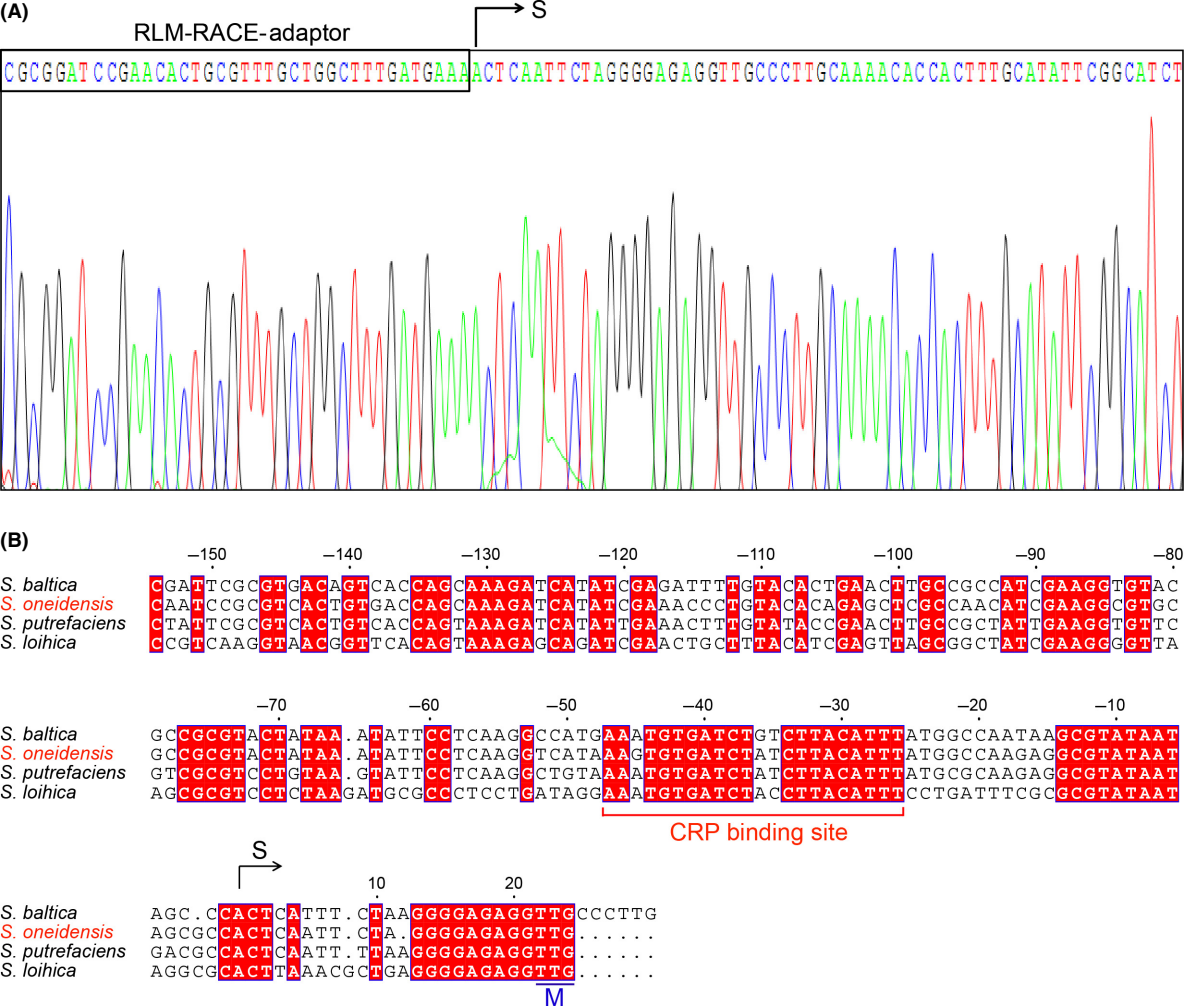
Use of 5′-RACE analyses to map the *Shewanella lipBA* promoter. (A) Direct DNA sequencing of the RLM-RACE product of the *Shewanella lipBA* operon. (B) Sequence comparison of the promoter regions of the *Shewanella lipBA* operon. The multiple alignments were conducted using ClustalW2 (http://www.ebi.ac.uk/Tools/clustalw2/index.html), and the resultant output was processed by program ESPript 2.2 (http://espript.ibcp.fr/ESPript/cgi-bin/ESPript.cgi) (Feng and Cronan 2009b, 2010; Feng et al. 2013a). Identical residues are indicated with white letters on a red background, similar residues are red letters on yellow, varied residues are in black letters, and dots represent missing residues. S, transcription start site; M, translational initiation site. The predicted CRP-recognizable palindrome is underlined. CRP, cAMP-receptor protein.

DNA sequences recognized by CRP proteins of E*. coli* and *S. oneidensis* are predicted to be similar and the interaction depends on cAMP (Gao et al. 2010; Fu et al. 2013; Zhou et al. 2013). Given the fact that a predicted CRP-binding site (AAGTGTGATCTATCTTACATTT) is located in the intergenic region between the *ybeD* gene and the *lipBA* operon of *S. oneidensis* (Fig.2), we thus mapped the promoter by employing an improved method of 5′-RACE (RLM-RACE). As a result, we acquired the 5′-RACE products of approximately 450 bp in length (Fig.3B). The result of the direct DNA sequencing showed the 5′-end of the *S. oneidensis lipBA* transcript (i.e., transcription start site, A) is located 20 nucleotides upstream its translation initiation codon TTG (Fig.4C and D). Apparently, the assumed CRP-recognizable site appears to be 25 bp upstream of the transcription start site (Fig.4D). Furthermore, the multiple sequence alignment clearly indicated that the CRP binding sites of different origins are extremely conserved, in that 16 of 22 nucleotides are identical at least (if not all) in the examined *Shewanella* species (Fig. 6A and B). However, the function of these putative sites needs further experimental validation.

### Physiological requirement of protein lipoylation

**Figure 5:**
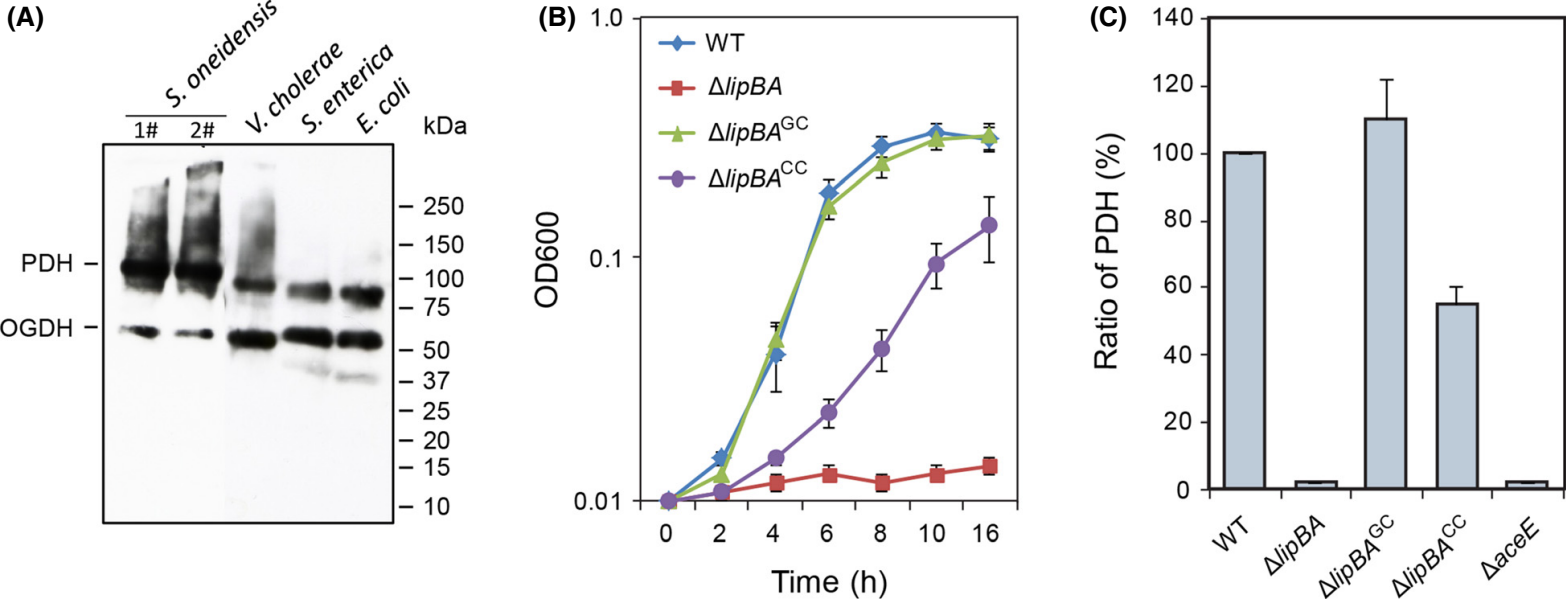
Physiological requirement of protein lipoylation in *γ*-proteobacteria. (A) Use of anti-LA Western blot to detect the requirement of protein lipoylation for *γ*-proteobacteria. Four species of *γ*-proteobacteria tested here include *Escherichia coli*, *Salmonella enterica* (*S. enterica*), *Vibrio cholerae* (*V. cholerae*) and *Shewanella oneidensis* (*S. oneidensis*). (B) Growth of the *S. oneidensis lipBA* mutant on lactate Complementation was carried out by either genetically (Δ*lipBA*^GC^, expressing a copy of the *lipBA* genes *in trans*) or chemically (Δ*lipBA*^CC^, with the addition of lipoic acid of 3 pmol/mL). (C) Analyses for PDH activity The PDH dehydrogenase activities are given as micromoles of 3-acetylpyridine adenine dinucleotide reduced per milligram of protein per hour for extracts of the same number of cells estimated by OD_600_ readings. The relative activities (RA) were obtained by normalizing the values of other strains to the mean of wild-type values. In both (B) and (C), error bars represent standard deviations from at least three independent experiments. PDH, pyruvate dehydrogenase; OGDH, 2-oxoglutarate dehydrogenase; LA, Lipoic acid; kDa, kilo-dalton.

It is reasonable that co-expression of LipB octanoyltransferase and LipA lipoate synthase assures the economical production of lipoic acid (an energy-expansive molecule) to effectively satisfy the metabolic/physiological requirement of protein lipoylation in organisms. Given the fact that both PDH and OGDH are proceeded such kind of post-translational modification, we thereby developed the anti-LA Western blot to detect this metabolic requirement in four γ-protebacteria species (*E. coli*, *S. enterica*, *V. cholerae,* and *S. oneidensis*). As expected, we did observe that lipoylation occurs in PDH and OGDH of *Shewanella*, which is in much similarity to the scenario seen with *E. coli* (Fig.5A). Because lipoylation is essential for the function of all characterized PDH and OGDH proteins, the result points out the metabolic significance of this common enzyme cofactor in *S. oneidensis*.

Subsequently, we constructed a *lipBA* null mutant from the *S. oneidensis* wild-type strain. The mutant was unable to grow on minimal medium unless lipoic acid was supplemented (Fig.5B), a phenotype observed from *E. coli lip* mutants (Reed and Cronan 1993). Additionally, the PDH assay revealed that this Δ*lipBA* strain contained no detectable dehydrogenase activities (Fig.5C). Importantly, the phenotypes resulting from the *lipBA* deletion were restored by their expression *in trans*, indicating that they are due to the intended mutation. These data, collectively, conclude that the *lipBA* genes are the only enzyme accountable for protein lipoylation in *S. oneidensis*.

### Characterization of *S. oneidensis* CRP protein

*S. oneidensis* CRP and its counterpart of *E. coli* are highly homologous (Fig. S1A), and have been shown to be functionally equivalent/exchangeable in vivo (Saffarini et al. 2003). However, whether this is the case in vitro remains undefined. In addition to the *E. coli* CRP protein, an N-terminal hexahistidine fused *S. oneidensis* CRP protein was over-expressed, purified to homogeneity and gave a single protein band with an estimated molecular mass (∽24 kDa) (Fig. S2B). The tertiary structure of *S. oneidensis* CRP protein was modeled using SPDBV_4.01 software, which is highly similar to that of *E. coli* (Fig. S3C). Liquid chromatography mass spectrometry analyses of tryptic peptides of the recombinant CRP protein band excised from an SDS-PAGE gel validated its identity in that the peptides matched *S. oneidensis* CRP with 70% coverage of the expected peptides (Fig. S4D). The two versions of CRP proteins we prepared were subsequently used for functional analyses of the above predicted CRP-specific palindromic sites.

### *Shewanella lipBA* binds the cAMP-CRP complex

**Figure 6:**
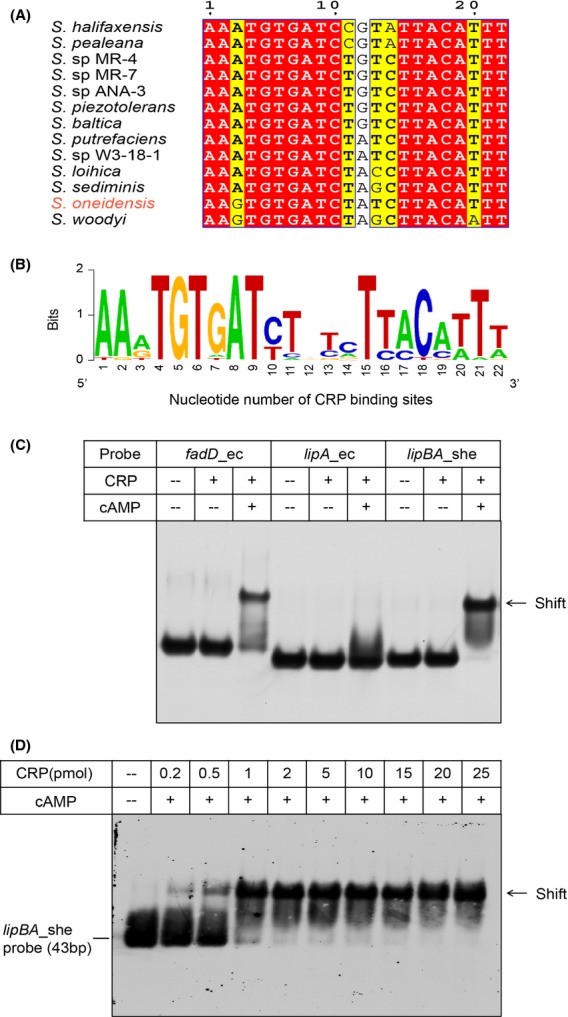
Binding of *Shewanella lipBA* to the cAMP-CRP functional complex. (A) Multiple sequence alignment of CRP-recognizable sites from *Shewanella lipBA* operon. Multiple sequence alignment was performed as described in Figure2. Identical residues are indicated with white letters on a red background, similar residues are black letters on yellow, and varied residues are in black letters. Totally, the CRP-binding sites are sampled from 13 different species of *Shewanella*. (B) Sequence logo for the CRP palindromic consensus sequences. The palindromic sequences used here are identical to those listed in (A), and the sequence logo was generated using WebLogo (http://weblogo.berkeley.edu/logo.cgi). (C) *Escherichia coli* CRP binds to *Shewanella lipBA* promoter, but not *E. coli lipA* promoter. The CRP site of *E. coli fadD* (*fadD*_ec) is used as positive control, while the possible CRP site of *E. coli lipA* (*lipA*_ec) is referred to negative control (Table2). The plus sign represents addition of the CRP protein and/or cAMP, whereas the minus sign denotes no addition of the CRP protein and/or cAMP. Designations: ec, *E. coli*; she, *Shewanella*. (D) Dose-dependent binding of *E. coli* CRP binds to *Shewanella lipBA* promoter. The level of CRP protein in (A) is 2 pmol, and the amount of cAMP is 20 pmol. The protein samples were incubated with 0.2 pmol of DIG-labeled *lipBA_*she probe (43 bp) in a total volume of 20 *μ*L. A representative result from three independent gel shift assays (7% native PAGE) is given. CRP, cAMP-receptor protein.

**Figure 7:**
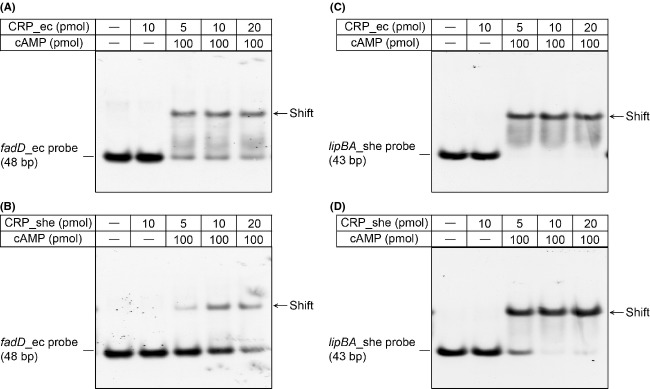
The two CRP proteins of *Escherichia coli* and *Shewanella* are functionally equivalent. (A) Binding of *E. coli* CRP to *E. coli fadD* probe. (B) *E. coli* CRP interacts with *Shewanella lipBA* promoter. (C) Interplay between *Shewanella* CRP and *E. coli fadD* probe. (D) *Shewanella* CRP binds *Shewanella lipBA* promoter. The CRP sites of *E. coli fadD* (*fadD*_ec) and *Shewanella lipBA* (*lipBA*_she) are listed in Table2. The plus sign denotes the addition of the CRP protein and/or cAMP, whereas the minus sign suggests no addition of the CRP protein and/or cAMP. Designations: ec, *E. coli*; she, *Shewanella*. When necessary in the EMSA tests, the level of cAMP is 20 pmol. The CRP protein samples were incubated with 0.2 pmol of DIG-labeled probe in a total volume of 20 *μ*L. A representative result is shown from three independent gel shift assays (7% native PAGE). CRP, cAMP-receptor protein.

To test the activity of the DNA probe derived from the *S. oneidensis lipBA* promoter (Fig.6A and B), we conducted EMSA assays. First, the positive control *fadD*_ec probe with a known function (Feng and Cronan 2012) binds well to *E. coli* CRP protein in the presence of cAMP effector molecule, whereas the negative control *lipA*_ec probe with a nonfunctional CRP site did not (Fig.6C). As expected, the *lipBA*_she probe exhibited the appreciably comparable activity of binding cAMP-CRP complex relative to the positive control. Apparently, our result is much consistent with previous observations with the CRP regulatory protein in the context of other metabolisms (Gao et al. 2010; Fu et al. 2013; Zhou et al. 2013), proving the prediction of Novichkov et al. (2013) is correct. Additionally, the specific binding of *lipBA*_she to cAMP-CRP complex seemed to be in a protein dose-dependent manner (Fig.6D).Not only does the CRP protein of *E. coli* origin interacts with *E. coli fadD* probe (Figs.6C and 7A) and *Shewanella lipBA* probe (Figs.6C and 7C), but also the CRP protein encoded by *Shewanella* binds to *E. coli fadD* probe (Fig.7B) and *Shewanella lipBA* probe (Fig.7D). It thus fully demonstrated that the two versions of CRP protein are functionally exchangeable in vitro.

Similarly, we also tested a series of predicted CRP-binding sites located upstream of *ybeD-lipB* loci (Fig.2 and Table3) using EMSA tests with *E. coli* CRP protein. Unlike the *lipBA*_she probe (Fig. S2A), neither the *E. coli ybeD* probe (*ybeD*_ec, Fig. S2B) nor the *Y. pestis ybeD* probe (*ybeD*_yp, Fig. S2G) are functional for the cAMP-CRP complex. By contrast, the prediction in CRP-recognizable sites (*ybeD*_es and *ybeD*_kp) in front of the *ybeD* gene of both *Enterobacter* sp. 638 and *Klebsiella pneumonia* are correct in that both bind to the CRP protein (Fig. S2C and D). Of particular note, among the two CRP sites (*ybeD*_st1 and *ybeD*_st2) proposed for *S. enteric ybeD* gene, only the *ybeD*_st1 site is functional (Fig. S2E), while the other one was not (Fig. S2F).

### A regulatory role for CRP in *lipBA* expression of *S. oneidensis*

**Figure 8:**
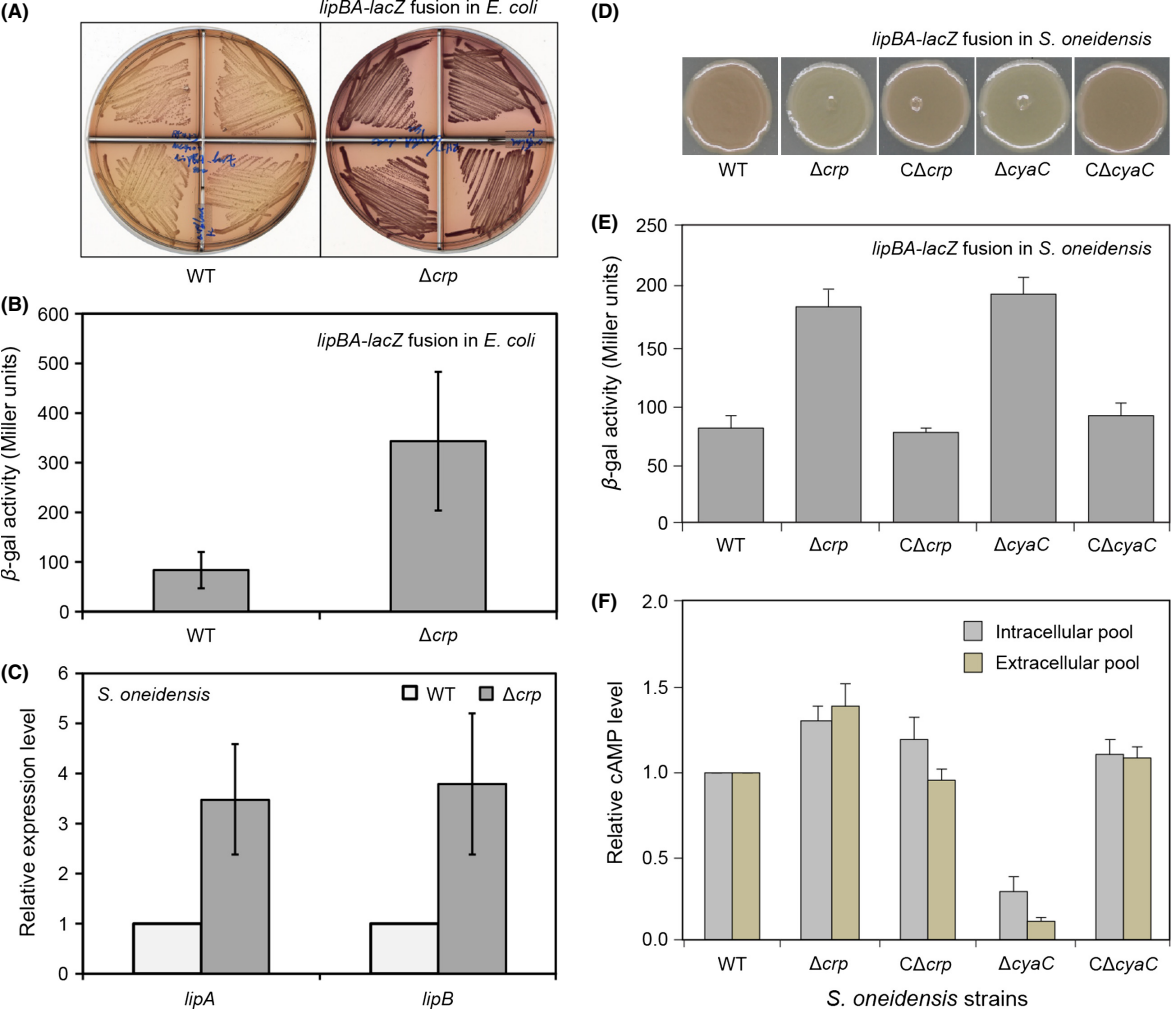
In vivo effect of CRP-cAMP complex on *lipBA* expression of *S. oneidensis*. (A) MacConkey agar plate-based visualization for effect of *Escherichia coli* CRP on *Shewanella lipBA* promoter-driven *lacZ* transcription. The two *E. coli* strains with the *lipBA-lacZ* transcriptional fusion include FYJ457 (WT) and FYJ458 (Δ*crp*). To assay *lipBA-lacZ* expression, we used MacConkey agar plate with 0.4% lactose as a sole carbon source. The bacteria were maintained at 37°C for around 36 h. Purple denotes high level of *β*-gal activity, whereas yellow indicates low level of *β*-gal activity. (B) *β*-gal analyses for CRP-mediated regulation of *lipBA*_she transcription in model organism *E. coli*. Mid-log phase cultures in RB media were collected to test *β*-gal activity. The data are expressed in average ± standard deviation (SD), and error bars indicate SD. No less than three independent experiments were performed. The two *E. coli* strains are FYJ457 (WT) and FYJ458 (Δ*crp*), respectively. (C) Real-time quantitative PCR (qPCR) assays for altered expression profile of *lipA* and *lipB* upon the removal of *crp* gene from *Shewanella*. The two strains of *Shewanella* grown in RB media are MR-1 (*S. oneidensis* MR-1, WT) and HG0624 (*S. oneidensis* MR-1, Δ*crp*). Mig-log phase bacteria were collected for isolation of total RNA. The data are expressed as averages ± standard deviations (SD), and error bars mean SD. Three independent experiments were performed here. Colony comparison (D) and *β*-gal activity (E) of the *S. oneidensis* reporter strains carrying the chromosomal *lipBA-lacZ* fusions grown on minimal medium plates. (F) Direct measurement of bacterial cAMP level. The intracellular (pelleted cells) and extracellular (supernatant) level of bacterial cAMP pools were assayed after centrifugation. A standard curve with cAMP by values of OD_450_ was generated for each patch of samples. Relative levels were calculated by normalizing to the values of the wild-type, which was set to 1. Both CΔ*crp* and CΔ*cya*C strains were designed to express a single copy of the corresponding genes in trans. Error bars represent standard deviations from at least three independent experiments. CRP, cAMP-receptor protein.

Two approaches (*lipBA*_she-*lacZ* transcriptional fusion and the real-time qRT-PCR) were used to examine the in vivo regulatory role of cAMP-CRP complex in expression of *S. oneidensis lipBA* operon encoding lipoic acid synthesis machinery. First, *S. oneidensis lipBA* promoter was fused to a LacZ reporter gene to allow direct assaying *β*-gal activity of *lipBA*_she-*lacZ* transcriptional fusion integrated into *E. coli* chromosome (Table1). In light of the functional equivalence of *Shewanella* CRP to *E. coli* CRP, we firstly compared the alteration of *β*-gal activity in the model organism *E. coli* (Δ*crp* mutant and its parental strain of *E. coli*). As anticipated, MacConkey plates-based experiments visualized that the *lipBA_*she promoter-driven *β*-gal activity is appreciably stronger (illustrated with purple) in the Δ*crp* mutant than that of the wild type *E. coli* (low activity denoted by yellow) (Fig.8A). Direct measurement of LacZ activity revealed that deletion of *crp* gene gave three- to fourfold increment of *lipBA_*she transcription level (Fig.8B). A similar *lacZ* reporter construct was also integrated into the chromosome of *S. oneidensis* wild-type and its Δ*crp* mutant strains (Fig.8D and E) (Fu et al. 2014). Consequently, the significant alteration/improvement of *lipBA_she-lacZ* expression level was detected upon the removal of the *crp* gene from *S. oneidensis* (Fig.8D and E). Second, the real-time qPCR-based analyses of transcriptional profile showed that no less threefold increment of *lipA* and/or *lipB* expression was observed in the Δ*crp* mutant of *S. oneidensis* in relative to the wild type strain (Fig.8C). Of particular note, repression of *S. oneidensis lipBA* expression by CRP depends on production of cyclic AMP (Fig.8E and F). Given the above combined in vitro and in vivo data, we concluded that the cAMP-CRP complex is a repressor for *lipBA* expression in *S. oneidensis*.

### Glucose improves the expression of *S. oneidensis lipBA* in the alternative model microorganism *E. coli*

It is well known that an addition of glucose into media can lower the level of cytosolic cAMP in *E. coli*, which might in turn impair at least partially CRP-mediated regulation. Somewhat it is unusual that not all the species of *Shewanella* genus can utilize/metabolize glucose in that the *S. oneidensis* glucose transporter-encoding gene *glcP* is a pseudo-gene with a frame-shift (Romine et al. 2008; Rodionov et al. 2010). Given the above technical problem, we therefore attempted to examine the so-called “glucose effect” with the engineered *E. coli* strain FYJ457 carrying the *lipBA*_she-*lacZ* transcriptional fusion (Fig. S3). As expected, we observed that the level of *lipBA* expression was induced by the addition of glucose (5 mmol/L) to about threefold higher than that grown in the M9 minimal media with acetate (5 mmol/L) as the sole carbon source (Fig. S3).

Together, we proposed for the first time that the global regulator, the cAMP-CRP complex represses bacterial lipoic acid synthesis in *Shewanella*, posing the relevance of the cAMP signaling to the production of the sulfur-containing C8 enzyme cofactor, lipoic acid (Fig.1A). This regulatory network can respond to the statue of glucose/cAMP level, i.e., the low glucose*/*high cAMP level shuts down *lipBA* expression (Fig.1B), whereas the high glucose*/*low cAMP level de-represses *lipBA* transcription (Fig.1C).

## Discussion

Biotin and lipoic acid both are sulfur-containing fatty acid derivatives and act as enzyme cofactors required for central metabolism in the three domains of life. Unlike the fact that the regulation of bacterial biotin metabolism has been extensively investigated, the knowledge about genetic control of lipoic acid synthesis remains missing or lagged. Although a recent bioinformatics-based proposal was raised, that is, the PDH repressor involved in *E. coli* lipoic acid synthesis (Kaleta et al. 2010; Gohler et al. 2011), this prediction was not validated by the physiological evidence in vivo (Feng and Cronan 2014). Through revisiting this long-term unanswered issue, the data shown here might establish for the first time the link of cAMP signaling to bacterial lipoic acid metabolism (Fig.1). Somewhat this finding can resolve the puzzle/discrepancy. As we knew that cAMP-dependent CRP regulatory system is a common global regulator involved in a variety of physiological processes (such as sugar metabolism) in most of the bacterial species, our finding extended this regulatory network into the field of vitamin synthesis.

As an important second messenger, the pool of cAMP molecule is at least determined by the following three factors: First, The activity of cyclic adenylate cyclase (*cyaA*) is responsible for the formation of cAMP molecules (Of note, 90% of cAMP that is made by intracellular adenylyl cyclases) (Pastan and Perlman 1970; Hantke et al. 2011); Second, the cAMP phosphodiesterase (CpdA) has the opposite enzymatic activity to break a phosphodiester bond of cAMP (Imamura et al. 1996). Not only is the production of CyaA regulated by the CRP regulator at the transcriptional level (Aiba 1985; Qu et al. 2013; You et al. 2013), but was also controlled at the metabolic level by the phosphorylation of EIIA^Glc^, a core component of PTS system (Crasnier-Mednansky 2008; Deutscher 2008; Gorke and Stulke 2008; Narang 2009). Third, the TolC pump is found to export cAMP outside of *E. coli* cells in maintaining its sensitivity in the changing metabolic environment (Hantke et al. 2011). Given the fact that all the three homologs of *cyaA* (SO_4312), *cpdA* (SO_3901), and *tolC* (SO_3904) are encoded in *Shewanella* genomes, it might raise the possibility of unexpected complexity in the linking of the second messenger cAMP signaling to lipoic acid synthesis in the context of *Shewanella* physiology. More interestingly, we recently discovered a novel enzyme, cAMP deaminase (referred to CadD) from the human pathogen *Leptospira interrogans* (Goble et al. 2013), and established a new mechanism for quenching the cAMP-dependent signaling. In light that we failed to search a CadD-like homolog, thereby we are not quite sure whether it might be implicated into bacterial lipoic acid synthesis or not yet.

The fact that two critical genes of lipoic acid synthesis *lipB* and *lipA* are organized into an operon (Figs.2 and 3), is somehow what we expected, in that it acts as a physiological advantage for economic and effective production of this enzyme cofactor. It is well-known that the genus *Shewanella* (belonging to the *γ*-proteobacteria) inhabited in energy-rich, redox-fluctuating environments and in turn evolved to possess diverse metabolic capabilities*,* e.g., coupling the turnover of organic matter with anaerobic respiration of different electron acceptors (Fredrickson et al. 2008). To our surprise, an appreciably conserved CRP-binding site is constantly present in the promoter regions of *lipBA* operon from nearly all the *Shewanella* species with sequenced genomes (Fig.2). It is of no doubt that a common and novel regulatory mechanism for lipoic acid biosynthesis is present in the genus *Shewanella*.

What kind of selective pressure or evolution consequence does it reflect in adaption to its unique environmental niche? In the paradigm microorganism *E. coli*, CRP is a global regulator for catabolite repression (Aiba 1983; Schultz et al. 1991; Chandler 1992). However, the protein in *S. oneidensis* was initially characterized as a principal regulator controlling anaerobic respiration of many electron acceptors (Saffarini et al. 2003). In recent years, it has been repeatedly shown that the regulator in fact plays a more comprehensive role in the physiology, covering both aerobic and anaerobic respiration (Gao et al. 2010; Dong et al. 2012; Fu et al. 2013, 2014; Zhou et al. 2013). In contrast to the pathway-specific regulators BirA (Beckett 2007) and BioR (Feng et al. 2013a,b), both of which negotiate production of the other enzyme cofactor biotin, we believed that *Shewanella* genus have evolved an unknown strategy to share the cAMP-dependent CRP regulatory architecture with other biological processes to efficiently control lipoic acid synthesis. Given the fact that glucose can induce *lipBA* expressions (Fig.1B), together with the above information, we concluded that the logic for this kind of regulation does make sense. The reasons are described as follows: (1) the anaerobic growth environment preferred by *Shewanella* determines an entry of glucose into the glycolytic pathway, giving two pyruvate molecules each glucose; (2) in the Krebs cycle, the resulting pyruvate is catalyzed by PDH to give acetyl-CoA; (3) the full activity of PDH requires the lipoylation, a post-translational modification of protein (which is validated by the scenario seen in the Anti-LA Western blot, i.e., PDH is the prevalent protein form relative to OGDH, Figure5); (4) the protein lipoylation depends on the availability of lipoic acids; (5) de novo LipB-LipA synthesis pathway is necessary to be turned on in addition to the LplA-mediated scavenging route of lipoic acid; (6) de-repression of *lipBA* expression might facilitate meeting the physiological requirement for lipoic acid production in such situation (vice versa, Fig.1C).

Of particular note, we also detected functional CRP-binding sites ahead of *ybeD* with unkown function in limited species such as human pathogen *S. enterica* (Figs.2 and S2). Although that *lipB* gene is adjacent to *ybeD* (of note, we lacked evidence proving if they are co-transcribed or not), it required further experimental evidence for CRP regulate *lipB* or not in this case. It is of interest to test this hypothesis. In fact, it has already been being our research direction in aiming to answer/pursue this question. To the best of our knowledge, our findings reveal, for the first time, a new molecular mechanism for genetic control of bacterial lipoic acid synthesis.

## Supporting Information

Figure S1 Characterization of Shewanella CRP protein. A. Sequence comparison of CRP proteins from three different organisms. As we described in Figures 2 and 4, the multiple alignments of CRP proteins were carried out using ClustalW2 (http://www.ebi.ac.uk/Tools/clustalw2/index.html). Identical residues are in white letters with red background, similar residues are in black letters with yellow background, varied residues arein black letters, and dots represent gaps. The predicted secondary structure was shown in top. *α*: *α*-helix; *β*: *β*-sheet; T: *β*-turns/coils. The three organisms used here are *E. coli*, *V. cholerae*, and *S. oneidensis*, respectively. (B) SDS-PAGE profile of the purified Shewanella CRP protein. The protein sample was separated with 4–20% gradient Mini-PROTEAN@ TGXTM Gel (Bio-Rad).The monomeric CRP protein with the estimated molecular weight of ∽24 kDa is indicated with an arrow. (C) Modeled structure of Shewanella CRP protein. Structure modeling was proceeded by the software of SPDBV_4.01 using *E. coli* CRP regulator with known structure (PDB: 2WC2) as structural template. N: N-terminus, C: C-terminus. (D) MS identification the recombinant Shewanella CRP protein. The peptide fragments that match Shewanella CRP protein are highlighted in bold and underlined type (70% coverage in total). **Figure S2.** Diversity in binding of bacterial ybeD probes to CRP protein. (A) The CRP site of Shewanella lipBA gene (referred to lipBA_she) can interact with *E. coli* CRP protein. (B) The predicted CRP site in front of *E. coli* ybeD-lipB-ybeF-lipA operon (ybeD_ec) has no ability to bind to the CRP protein. The putative CRP sites of the ybeD-lipB-ybeF-lipA operon from Enterobacter sp. 638 (ybeD_es, C) and Klebsiella pneumonia (ybeD_kp, D) are functional. The predicted CRP site 1 of Salmonella enteric ybeD-lipB-ybeF-lipA operon is functional (E), whereas the site 2 is inactive (F). (G) No binding of the cAMP-CRP complex to the suspected CRP site in front of the ybeD-lipB-lipA operon of Yersinia pestis. All the EMSA experiments (7% native PAGE) were conducted as we described (Feng and Cronan 2012; Feng et al. 2013a) with a minor change. The level of cAMP added is 20 pmol. The *E. coli* CRP protein samples in various concentrations were incubated with 0.2 pmol of DIG-labeled probe in a total volume of 15 *µ*L. A representative result is given. The sequences of all the DNA probes used here are listed in Tables 2 and 3. The minus sign denotes no addition of the CRP protein and/or cAMP molecule. Designations: she, Shewanella; ec, *E. coli*; es, Enterobacter sp. 638; kp, Klebsiella pneumonia; st, Salmonella typhimurium LT2, and yp,Yersinia pestis. **Figure S3.** Induction of Shewanella lipBA expression by glucose in the alternative model *E. coli*. To test the effect of glucose on Shewanella lipBA expression, the *E. coli* strain carrying the lipBA_she-lacZ transcriptional fusion (FYJ457) was used here. Mid-log phase cultures in M9 media with acetate and/or glucose (5 mmol/L) as sole carbon source were sampled for assaying *β*-gal activity. The data from more than three independent experiments is expressed in average ± standard deviation (SD), and error bars indicate SD.
