# Supplementary material for: A new regulatory mechanism for bacterial lipoic acid synthesis
Source: Microbiologyopen. 2015 Jan 22;4(2):282–300. doi: 10.1002/mbo3.237 (PMC4398509; doi:10.1002/mbo3.237)
Supplement: Figure S1 — Characterization of Shewanella CRP protein. A. Sequence comparison of CRP proteins from three different organisms. As we described in Figures 2 and 4, the multiple alignments of CRP proteins were carried out using ClustalW2 (http://www.ebi.ac.uk/Tools/clustalw2/index.html). Identical residues are in white letters with red background, similar residues are in black letters with yellow background, varied residues arein black letters, and dots represent gaps. The predicted secondary structure was shown in top. α: α-helix; β: β-sheet; T: β-turns/coils. The three organisms used here are E. coli, V. cholerae, and S. oneidensis, respectively. (B) SDS-PAGE profile of the purified Shewanella CRP protein. The protein sample was separated with 4–20% gradient Mini-PROTEAN@ TGXTM Gel (Bio-Rad).The monomeric CRP protein with the estimated molecular weight of ∽24 kDa is indicated with an arrow. (C) Modeled structure of Shewanella CRP protein. Structure modeling was proceeded by the software of SPDBV_4.01 using E. coli CRP regulator with known structure (PDB: 2WC2) as structural template. N: N-terminus, C: C-terminus. (D) MS identification the recombinant Shewanella CRP protein. The peptide fragments that match Shewanella CRP protein are highlighted in bold and underlined type (70% coverage in total). Figure S2. Diversity in binding of bacterial ybeD probes to CRP protein. (A) The CRP site of Shewanella lipBA gene (referred to lipBA_she) can interact with E. coli CRP protein. (B) The predicted CRP site in front of E. coli ybeD-lipB-ybeF-lipA operon (ybeD_ec) has no ability to bind to the CRP protein. The putative CRP sites of the ybeD-lipB-ybeF-lipA operon from Enterobacter sp. 638 (ybeD_es, C) and Klebsiella pneumonia (ybeD_kp, D) are functional. The predicted CRP site 1 of Salmonella enteric ybeD-lipB-ybeF-lipA operon is functional (E), whereas the site 2 is inactive (F). (G) No binding of the cAMP-CRP complex to the suspected CRP site in front of the ybeD-lipB-lipA operon of Yers [file mbo30004-0282-sd1.docx]

**Supplemental figures**


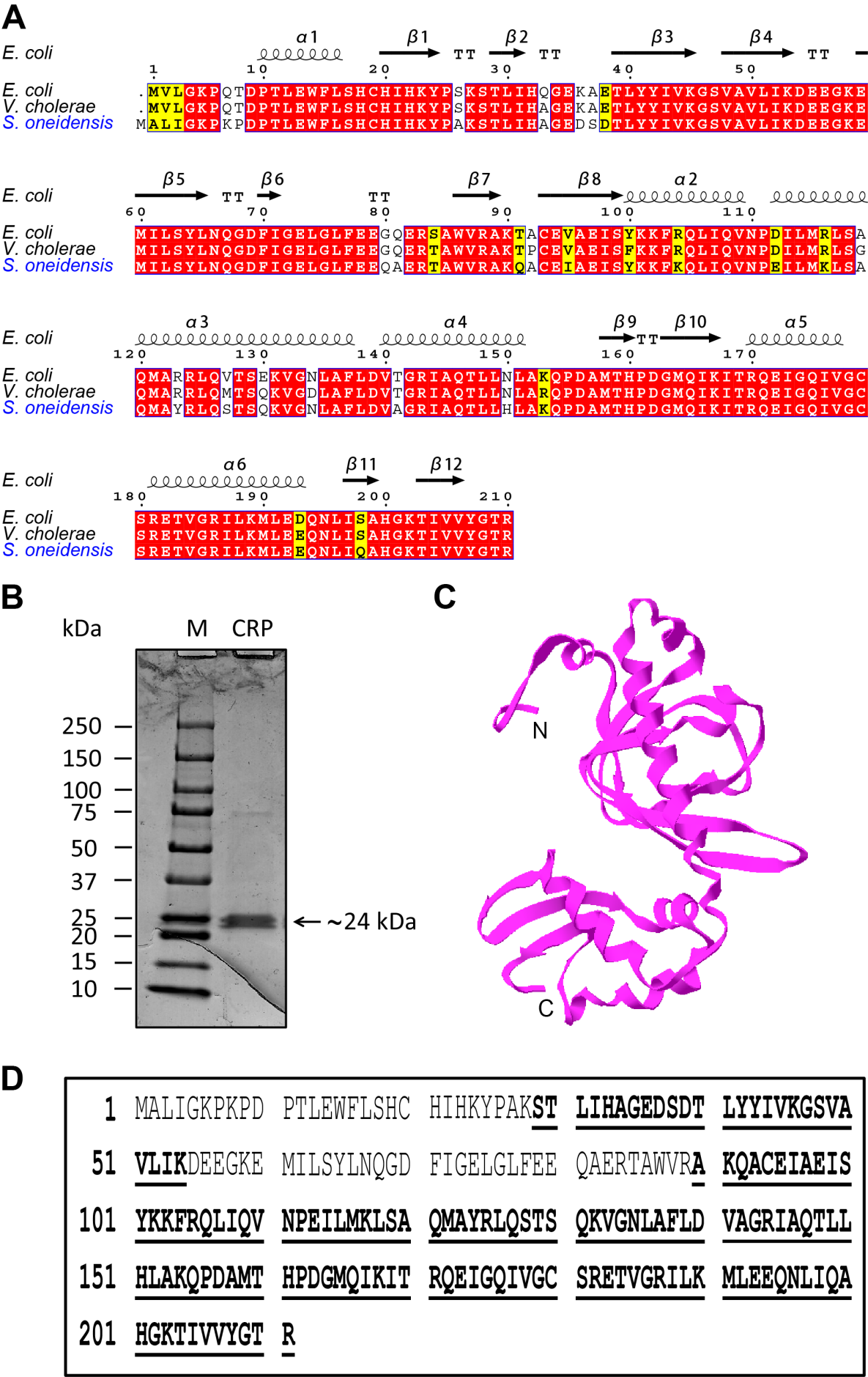


**Figure S1** Characterization of *Shewanella* CRP protein

**A.** Sequence comparison of CRP proteins from three different organisms

As we described in **Figure 2** and **4**, the multiple alignments of CRP proteins were carried out using ClustalW2 (<http://www.ebi.ac.uk/Tools/clustalw2/index.html>). Identical residues are in white letters with red background, similar residues are in black letters with yellow background, varied residues arein black letters, and dots represent gaps. The predicted secondary structure was shown in top. α: α-helix; β: β-sheet; T: β-turns/coils. The three organisms used here are *E. coli*, *V. cholerae* and *S. oneidensis*, respectively.

**B.** SDS-PAGE profile of the purified *Shewanella* CRP protein

The protein sample was separated with 4-20% gradient Mini-PROTEAN@ TGXTM Gel (Bio-Rad).The monomeric CRP protein with the estimated molecular weight of ~ 24 kDa is indicated with an arrow.

**C.** Modeled structure of *Shewanella* CRP protein

Structure modeling was proceeded by the software of SPDBV_4.01 using *E. coli* CRP regulator with known structure (PDB: 2WC2) as structural template. N: N-terminus, C: C-terminus.

**D.** MS identification the recombinant *Shewanella* CRP protein

The peptide fragments that match *Shewanella* CRP protein are highlighted in bold and underlined type (70% coverage in total).­­­





**Figure S2** Diversity in binding of bacterial *ybeD* probes to CRP protein

1. The CRP site of *Shewanella lipBA* gene (referred to *lipBA*_she) can interact with *E. coli* CRP protein
2. The predicted CRP site in front of *E. coli ybeD-lipB-ybeF-lipA* operon (*ybeD*_ec) has not ability to bind CRP protein

The putative CRP sites of the *ybeD-lipB-ybeF-lipA* operon from *Enterobacter* sp. 638 (*ybeD*_es, **Panel C**) and *Klebsiella pneumonia* (*ybeD*_kp, **Panel D**) are functional.

The predicted CRP site 1 of *Salmonella enteric ybeD-lipB-ybeF-lipA* operon is functional (**Panel E**), whereas the site 2 is inactive (**Panel F**).

**G.** No binding of the cAMP-CRP complex to the suspected CRP site in front of the *ybeD-lipB-lipA* operon of *Yersinia pestis*

All the EMSA experiments (7% native PAGE) were conducted as we described ([Feng and Cronan, 2012](#_ENREF_17); [Feng et al., 2013a](#_ENREF_20)) with minor change. The level of cAMP added is 20 pmol. The *E. coli* CRP protein samples in various concentrations were incubated with 0.2 pmol of DIG-labeled probe in a total volume of 15 µl. A representative result is given.

The sequences of all the DNA probes used here are listed in **Table 2** and **3.**

The minus sign denotes no addition of the CRP protein and/or cAMP molecule. Designations: she, *Shewanella*; ec, *E. coli*; es, *Enterobacter* sp. 638; kp, *Klebsiella pneumonia*; st, *Salmonella typhimurium* LT2, and yp,*Yersinia pestis*.





**Figure S3** Induction of *Shewanella lipBA* expression by glucose in the alternative model *E. coli*

To test effect of glucose on *Shewanella lipBA* expression, the *E. coli* strain carrying the *lipBA*_she*-lacZ* transcriptional fusion (FYJ457) was used here. Mid-log phase cultures in M9 media with acetate and/or glucose (5 mM) as sole carbon source were sampled for assaying β–gal activity. The data from more than three independent experiments is expressed in Average ± standard deviation (SD), and error bars indicate SD.
